# Mechanisms of PDGF siRNA-mediated inhibition of bone cancer pain in the spinal cord

**DOI:** 10.1038/srep27512

**Published:** 2016-06-10

**Authors:** Yang Xu, Jia Liu, Mu He, Ran Liu, Visar Belegu, Ping Dai, Wei Liu, Wei Wang, Qing-Jie Xia, Fei-Fei Shang, Chao-Zhi Luo, Xue Zhou, Su Liu, JohnW. McDonald, Jin Liu, Yun-Xia Zuo, Fei Liu, Ting-Hua Wang

**Affiliations:** 1 Institute of Neurological Disease, Department of Anesthesiology and Translation Neuroscience Center, West China Hospital, Sichuan University, Chengdu 610041, PR China; 2 Institute of Neuroscience, Kunming Medical University, Kunming 650031, PR China; 3 Department of Neurosurgery, West China Hospital, Sichuan University, Chengdu 610041, PR China; 4 Department of Neurology, Johns Hopkins School of Medicine, Baltimore, MD, USA, International Center for Spinal Cord Injury, Hugo W. Moser Research Institute at Kennedy Krieger Inc., Baltimore, MD, USA; 5 Department of Histology, Embryology and Neurobiology, West China School of Preclinical and Forensic Medicine, Sichuan University, Chengdu, Sichuan 610041, China

## Abstract

Patients with tumors that metastasize to bone frequently suffer from debilitating pain, and effective therapies for treating bone cancer are lacking. This study employed a novel strategy in which herpes simplex virus (HSV) carrying a small interfering RNA (siRNA) targeting platelet-derived growth factor (PDGF) was used to alleviate bone cancer pain. HSV carrying PDGF siRNA was established and intrathecally injected into the cavum subarachnoidale of animals suffering from bone cancer pain and animals in the negative group. Sensory function was assessed by measuring thermal and mechanical hyperalgesia. The mechanism by which PDGF regulates pain was also investigated by comparing the differential expression of pPDGFRα/β and phosphorylated ERK and AKT. Thermal and mechanical hyperalgesia developed in the rats with bone cancer pain, and these effects were accompanied by bone destruction in the tibia. Intrathecal injection of PDGF siRNA and morphine reversed thermal and mechanical hyperalgesia in rats with bone cancer pain. In addition, we observed attenuated astrocyte hypertrophy, down-regulated pPDGFRα/β levels, reduced levels of the neurochemical SP, a reduction in CGRP fibers and changes in pERK/ERK and pAKT/AKT ratios. These results demonstrate that PDGF siRNA can effectively treat pain induced by bone cancer by blocking the AKT-ERK signaling pathway.

Cancer patients with bone metastases suffer from debilitating pain that severely reduces their quality of life^1^,^2^. The World Cancer Report 2014 revealed that 14.1 million new cancer cases were diagnosed and 8.2 million people died from cancer^3^. Among patients with terminal breast and prostate cancer, 70% develop neoplastic bone metastases^4^,^5^. Osseous metastasis pain associated with malignant tumors (cancer induced bone pain, CIBP) is a complex state comprising background pain, spontaneous pain and evoked pain^6^,^7^. Approximately 75% of cases of bone cancer pain include spontaneous pain and evoked pain, and more than 40% of these patients experience unpredictable bursts of pain^8^,^9^,^10^. Current therapeutic strategies for alleviating cancer pain are often inadequate and are associated with multiple side effects listed in the guidelines of the WHO analgesic ladder^6^,^11^. Therefore, new approaches for treating bone cancer pain and the underlying mechanisms mediating bone cancer pain warrant further investigation^12^,^13^.

Gene therapy enables the correction of inherited gene aberrations^14^,^15^ and represents a novel and targeted approach for treating pain at the level of the spinal cord^16^,^17^. Herpes simplex virus (HSV), a member of the human herpes virus family, is particularly well suited for gene delivery to the peripheral nervous system (PNS) due to its natural affinity for PNS neurons. HSV can carry 30–50 kb of foreign DNA and can reside in a broad range of host cell types, and its genome can be maintained as an episomal element in non-dividing cells^16^,^18^,^19^. HSV-mediated gene therapy has been employed to correct genetic defects associated with proenkephalin, glutamic acid decarboxylase, anti-inflammatory peptides and glial cell-derived neurotrophic factor. Correcting aberrations in these genes can partially relieve pain^16^,^20^, indicating that HSV-based constructs carrying siRNA have the potential to effectively treat bone cancer pain.

Platelet-derived growth factor (PDGF), a cationic disulfide-linked polypeptide that exists as a homo- or heterodimer, can promote cell migration, proliferation, and differentiation^21^,^22^. PDGF is involved in several pathologies, including oncogenesis, atherosclerosis, and lung and kidney fibrosis^23^,^24^,^25^. Moreover, hyper-active PDGF results in tactile allodynia, and intrathecal administration of either a selective inhibitor of PDGF receptor (PDGFR) phosphorylation or an antibody blocking endogenous PDGF suppresses thermal hyperalgesia and tactile allodynia^26^,^27^. These results suggest that PDGF knockdown might relieve mechanical and thermal hyperalgesia.

In the present study, we developed a clinically relevant model of bone cancer pain and investigated the potential role of PDGF in mediating bone cancer pain. An HSV vector carrying 2 artificial PDGF small interfering RNAs was intrathecally injected into the L4–6 spinal cord segments of animals with bone cancer pain. The injection of tumor cells into the tibia induced an increase in PDGF levels in the L4–6 spinal segment. In addition, immunofluorescence staining demonstrated that PDGF localized to microglia cells, astrocytes and neurons, cell types expected to be infected by HSV. These data demonstrate that reducing PDGF expression using RNAi inhibits the expression of glial fibrillary acidic protein (GFAP), substance p (SP), calcitonin gene-related peptide (CGRP) and the phosphorylation of ERK and AKT and therefore represents a novel strategy for treating bone cancer pain.

## Results

### Anatomical and behavioral characterization of the bone cancer pain model

**Figure 1:**
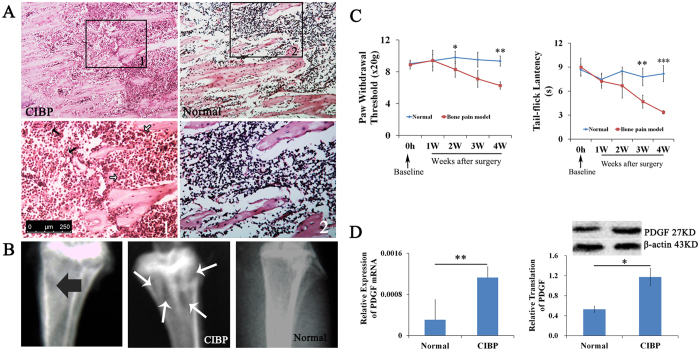
Bone cancer-induced bone destruction, thermal and mechanical hyperalgesia, and increased PDGF expression. **(A)** Hematoxylin and eosin (H&E) staining of the tibia revealed that bone marrow spaces were infiltrated with malignant tumor cells (black arrows) and osteoclasts (white arrows) on day 28 after the injection of tumor cells. The images in the lower panel are magnified images of the boxes in the images in the upper panel. **(B)** High-resolution radiographs of rat hind limbs after the bilateral injection of tumor cells into the rostral part of the tibia. The left image indicates the site of tumor cell injection (black arrow), and the middle image indicates areas of bone destruction (white arrows). **(C)** Paw withdrawal threshold and tail-flick latency were evaluated over the indicated time course. **(D)** Animals with bone cancer pain exhibited elevated expression levels of PDGF as measured by qPCR and western blot. Data are presented as the mean ± SEM. ^*^P < 0.05, ^**^P < 0.01, ^***^P < 0.001.

Histological and radiological analyses were performed to confirm tumor formation following the injection of tumor cells into the bilateral tibia in the bone cancer pain model. Hematoxylin and eosin (H&E) staining 28 days after injection demonstrated that the bone matrix in the bone marrow of these animals was damaged and filled with tumor cells and osteoclasts; these features were not observed in the normal control rats (Fig. 1A). Furthermore, radiographs of the animals’ hind limbs revealed bone destruction in the bilateral tibia compared with the control rats (Fig. 1B).

To evaluate the development of pain-related behaviors induced by the intra-tibial injection of tumor cells, tail-flick latency (TL) and paw withdrawal threshold (PWT) were assessed at baseline and 1, 2, 3 and 4 weeks after tumor cell inoculation. The animals in the bone cancer pain group exhibited a decrease in TL or PWT over time. There was a significant decrease in TL and PWT 4 weeks after tumor cell injection (Fig. 1C). The decrease in TL was observed as early as 21 days after the injection of tumor cells (P = 0.001) (Fig. 1C) and worsened over the time course of experiment (Fig. 1C). The decrease in TL following thermal stimulation indicated the development of thermal hyperalgesia in the bone cancer pain rat model.

### Bone cancer pain enhances PDGF expression in neurons and glia cells in the spinal cord

**Figure 2:**
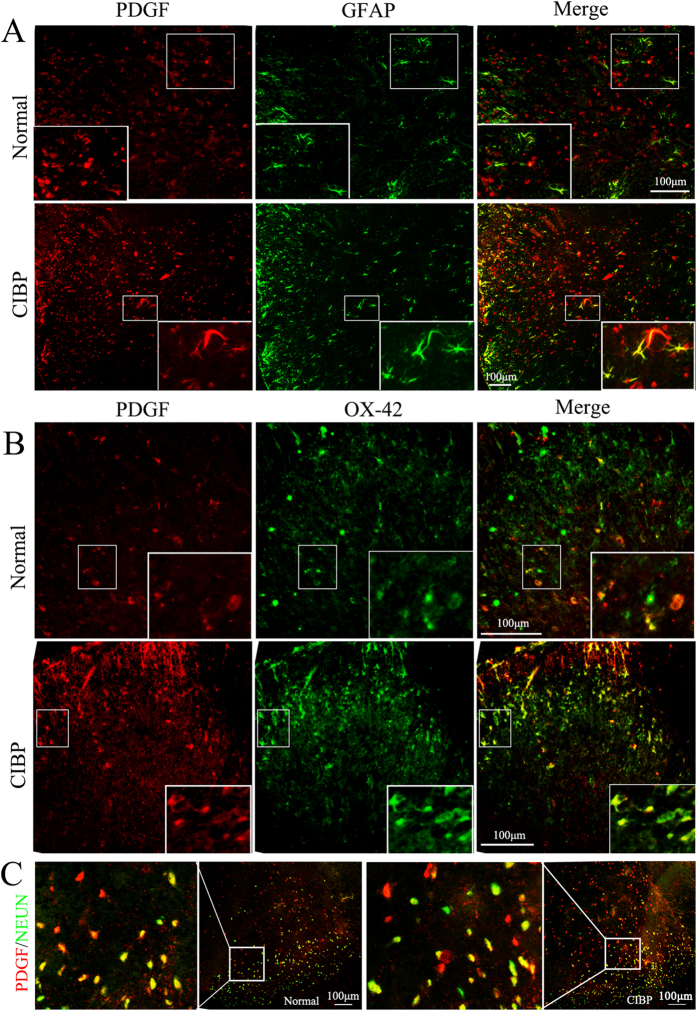
Enhanced expression of PDGF in neurons, astrocytes and microglia cells in the spinal cords of rats with bone cancer pain compared with normal rats. (**A,B,C**) Double immunofluorescence labeling in the CIBP and normal groups demonstrated that PDGF (red) localized to neurons, astrocytes and microglia cells in the dorsal horn of the spinal cord. The inset of each image is a magnified view of the indicated region. NEUN: neuron marker (green); GFAP: astrocyte marker (green); OX-42: microglia maker (green).

To investigate the molecular mechanisms by which bone cancer induces pain, we examined the expression of PDGF in the spinal cord. Changes in the levels of PDGF in animals with bone cancer pain were detected at the mRNA and protein levels. PDGF mRNA expression was elevated in animals with bone cancer pain (0.00113 ± 0.000204) compared with the control rats (0.00031 ± 0.000390) (P = 0.002) (Fig. 1D). Consistent with these findings, PDGF protein levels were elevated in the bone cancer pain group (1.17 ± 0.174790) compared with the control group (0.53 ± 0.060825) (P = 0.004) (Fig. 1D). Immunofluorescence staining demonstrated that PDGF was expressed in neurons, astrocytes and microglia cells (Fig. 2). The number of cells expressing PDGF and the astrocyte marker GFAP increased in the rats with CIBP compared with the control rats (Fig. 2A). Similar results were observed for staining of the microglia cell marker OX-42 and PDGF (Fig. 2B). The number of NeuN-positive cells should remain unchanged if neuronal cell death or neurogenesis does not occur. No detectable changes in the number of neurons were observed, but there was an increase in PDGF-positive cells in the rats with CIBP compared with the control rats (Fig. 2C).

### Characterization of the HSV-1-PDGF siRNA vector

**Figure 3:**
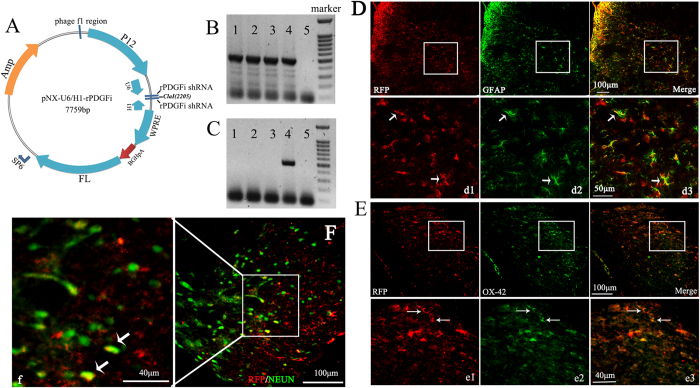
The PDGF siRNA replication-incompetent HSV-1 vector. **(A)** Schematic representation of the HSV-1-based gene transfer vector. The construct was created by deleting the 3 immediate-early genes (ICP4, ICP27 and ICP34.5) of wild-type HSV-1. The vector also contained the human cytomegalovirus (CMV) promoter, WPRE and a polyA (pA) region. **(B)** The presence of the desired elements in the vector, denoted pNX01-U6H1-rPDGFi, was confirmed by RT-PCR analysis. Markers (100 bp DNA ladder, from top to bottom): 1,500, 1,000, 900, 800, 700, 600, 500, 400, 300, 200 and 100 bp fragments. **(C)** The production of NX01-U6H1-rPDGFi was confirmed by RT-PCR analysis. **(D–F)** Cells infected with the HSV virus were analyzed by double immunofluorescence labeling 7 days after the intrathecal injections. Panels d1–d3, e1–e3 and f are magnified views of the boxes in D–F, respectively, and demonstrate the colocalization for RFP (red) with GFAP (green), OX-42 (green) and NEUN (green). RFP is a marker protein in the HSV vector.

To investigate the effect of increased levels of PDGF in rats with CIBP, we constructed a replication-incompetent HSV-1 vector carrying a siRNA to inhibit PDGF gene expression via RNA interference (RNAi) (Fig. 3A). Semi-quantitative RT-PCR analysis using the U6-F/H1-F primer demonstrated that pNX01-U6H1-rPDGFi vector samples 1–4 contained the approximately 477-bp PDGF siRNA sequence (Fig. 3b). Sample 4 of the virus of NX01-U6H1-rPDGFi also included the target sequence of the PDGF siRNA as demonstrated by semi-quantitative RT-PCR analysis using the same primer (Fig. 3C). The infection efficiency of the HSV virus was evaluated by immunofluorescence assays 7 days following the injection of the viruses into the rat spinal cords. The red signal in Fig. 3 represents red fluorescent protein (RFP), the marker used to label the HSV vector. Cells throughout the dorsal horn of the spinal cord including astrocytes, microglia cells and neurons, were infected with the virus as demonstrated by the colocalization of RFP and cell type-specific markers (green; Fig. 3D–F).

### Inhibiting PDGF expression attenuates tumor-evoked mechanical and thermal hyperalgesia

**Figure 4:**
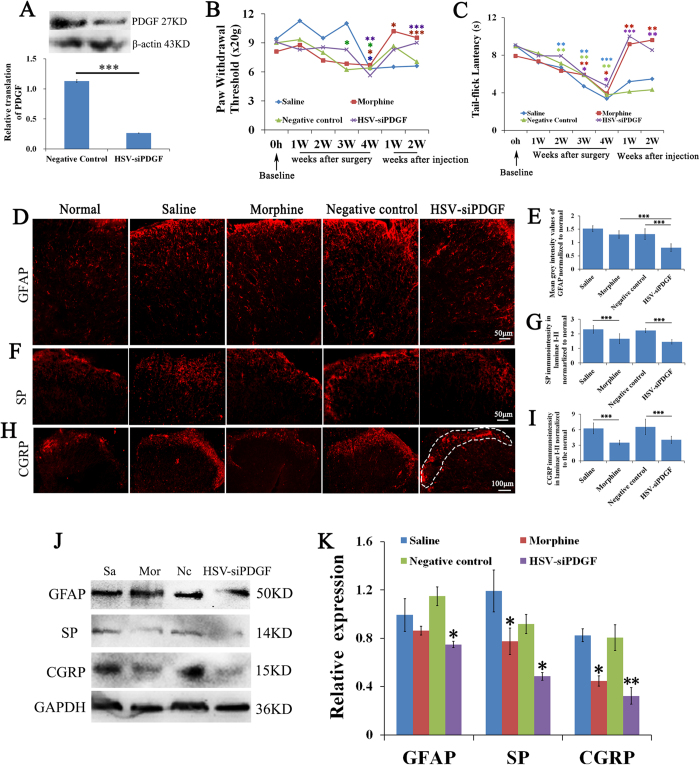
PDGF siRNA down regulates PDGF protein levels, attenuates tumor-evoked mechanical and thermal hyperalgesia, reverses bone cancer pain-evoked astrocyte hypertrophy and reduces the expression of substance P (SP) and CGRP in laminae I-II of L4–6 in the dorsal horn of the spinal cord. (**A**) Treatment with PDGF siRNA down-regulated the transcription of PDGF. (**B**) Paw withdrawal thresholds for each treatment groups were evaluated throughout the study. (**C**) The tail-flick latencies of each treatment group were evaluated throughout the study. (**D,F,H**) Images of the dorsal horn showing the distribution of the astrocyte markers GFAP, SP and CGRP in transverse sections at spinal cord segments L4–L6 in animals that received saline, morphine, negative control siRNA and PDGF siRNA. (**E**) Quantification of GFAP immunofluorescence demonstrated that treatment with PDGF siRNA reduced GFAP-immunoreactivity in tumor-injected rats. (**G,I**) Quantification of SP and CGRP in laminae I-II revealed a reduction following treatment with PDGF siRNA or morphine. DAPI: nuclear marker (blue). The white dashed area shows laminae I-II of the spinal cord. (J, K) The protein levels of GFAP, SP and CGRP were consistent with the results of immunofluorescence. Symbols with different colors represent different groups. Data are presented as the mean ± SEM. ^*^P < 0.05, ^**^P < 0.01, ^***^P < 0.001.

Treatment with PDGF siRNA reduced PDGF protein expression in the L4–L6 spinal cord segments of animals with CIBP compared with the negative control treatment (P = 0.000) (Fig. 4A). The second week following tumor cell inoculation, the rats with CIBP exhibited an increase in thermal hyperalgesia from baseline (blue and green asterisk in Fig. 4C), whereas 7 days after the injection of the PDGF siRNA, thermal hyperalgesia was attenuated in the treated animals compared with the negative control vector (NC)-treated animals (P = 0.000) (purple asterisk, Fig. 4C). Fourteen days after the injection of PDGF siRNA in L4-L6, we observed improvements in PWT and TL compared with the NC-treated group (P = 0.000 and P = 0.002, respectively) (purple asterisk, Fig. 4B,C). Treatment with morphine also attenuated mechanical and thermal hyperalgesia 7 days after administration compared with saline treatment (P = 0.012 and P = 0.000, respectively, for PWT; P = 0.002 and P = 0.004 for TL, respectively) (red asterisk, Fig. 4B,C). PDGF siRNA was as effective as morphine in alleviating mechanical and thermal hyperalgesia.

### PDGF down-regulation and treatment with morphine reverses bone cancer pain-induced changes in the expression of the neurochemicals GFAP, SP and CGRP

To investigate the mechanisms by which PDGF induces bone cancer pain, we measured changes in the levels of the neurochemicals SP and CGRP and evaluated astrocyte hypertrophy in the spinal cord. Rats injected with tumor cells exhibited increased levels of SP in laminae I-II, and the RNAi-induced reduction of PDGF levels suppressed SP levels in animals with CIBP (1.4 ± 0.1575) compared with the NC-treated animals (2.2 ± 0.1452) (P = 0.000, Fig. 4F,G). In addition, SP immunoreactivity decreased by 1.7 ± 0.3434 in animals treated with morphine compared with animals treated with saline (P = 0.000) (Fig. 4F,G). These results are consistent with the inhibition of SP protein expression in the spinal cord by PDGF down-regulation in the bone cancer pain group compared with the control group (P = 0.025). Morphine treatment also reduced the expression of SP compared with saline treatment (P = 0.029) (Fig. 4J,K).

Injection of tumor cells into the tibia also increased CGRP-immunoreactivity in the laminae I-II of spinal cord segments L4–L6, whereas PDGF siRNA (4.1 ± 0.6930) suppressed the enhanced CGRP-immunoreactivity compared with NC siRNA (P = 0.000) (Fig. 4H,I). Morphine administration also reduced CGRP- immunoreactivity (3.5 ± 0.4512) compared with saline treatment (P = 0.000) (Fig. 4H,I). Similar results were obtained by western blot (WB) analysis, which revealed that PDGF siRNA down-regulated CGRP expression compared to the NC-treated group (P = 0.009) (Fig. 4J,K) and that morphine treatment reduced CGRP protein levels compared to saline treatment (P = 0.037) (Fig. 4J,K).

In addition to the increase in neurochemicals, we also observed an increase in astrocyte hypertrophy (an indicated by increased GFAP-immunoreactivity) in the L4-L6 spinal segments of animals with CIBP. PDGF siRNA reduced GFAP immunofluorescence (0.81 ± 0.1417) compared with NC siRNA and morphine (P = 0.000 for both) (Fig. 4D,E). Morphine treatment, however, did not influence GFAP immunofluorescence (Fig. 4D,E). In addition, treatment with PDGF siRNA reduced GFAP expression compared with the NC control (P = 0.049) (Fig. 4J,K).

### Reducing PDGF expression inhibits the phosphorylation of PDGF receptors and suppresses pain induced ERK and AKT signaling

**Figure 5:**
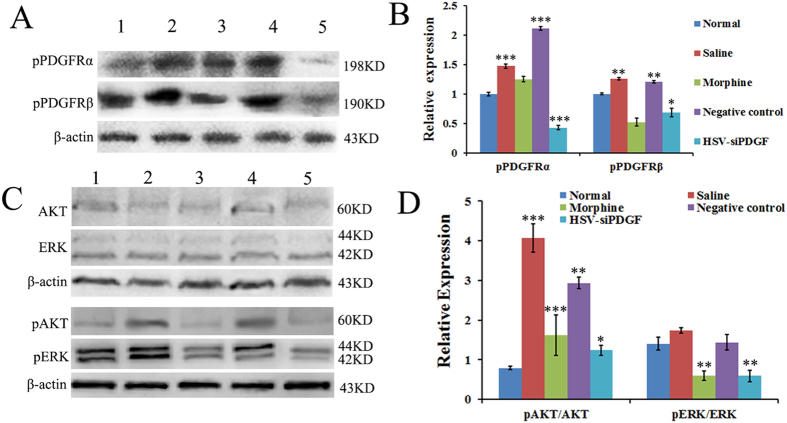
Bone cancer pain increases phosphorylation of PDGFRs, ERK and AKT in the dorsal horn of spinal cord L4–6 segments, and PDGF siRNA reverses this effect. (**A–D**) Western blot was performed to evaluate the levels of phosphorylated PDGFRs (**A,B**), AKT and extracellular regulated kinase (ERK)1/2 (**C,D**) in protein extracts from the L4–6 segment of the spinal cord in each group. The level of pPDGFRs decreased in the PDGF siRNA-treated group (**A,B**). Treatment with PDGF siRNA and morphine significantly reduced the pERK/ERK and pAKT/AKT ratios (**C,D**). β-actin, a house-keeping gene was used as an internal control. Lane 1: Normal; Lane 2: Treatment with saline; Lane 3: Treatment with morphine; Lane 4: Treatment with negative control siRNA; Lane 5: Treatment with PDGF siRNA. Data are represented as the mean ± SEM. ^*^P < 0.05, ^**^P < 0.01, ^***^P < 0.001.

In an effort to identify the downstream mechanisms by which PDGF induces bone cancer pain, we used WB to evaluate the activation (phosphorylation) of PDGFR, ERK and AKT by measuring levels of pPDGFRα/β, pERK and pAKT 14 days after treatment with PDGF siRNA. The increase in PDGF expression in the spinal cord segments L4–6 of mice injected with tumor cells coincided with increases in pPDGFRα/β and pAKT/AKT expression compared with the control group (Fig. 5A,B). As shown in histogram B, pPDGFRα/β levels increased in the saline- and NC- treated groups compared with the normal group (P = 0.000 for both in the left illustration and P = 0.001, 0.002 for the right, respectively), whereas histogram C demonstrates that pAKT/AKT levels were elevated in the saline- and NC-treated groups compared with the normal group (P = 0.001 and P = 0.002, respectively). pPDGFRα/β levels decreased in the HSV-siPDGF-treated group compared with the NC siRNA-treated group (P = 0.000 and P = 0.017, respectively). A similar trend was observed in the morphine-treated group but was not statistically significant. PDGF siRNA treatment reduced the pERK:ERK and pAKT:AKT ratios compared with NC siRNA treatment (P = 0.01 and P = 0.09, respectively) (Fig. 5C,D). Morphine treatment also reduced the pERK:ERK and pAKT:AKT ratios compared with saline treatment (P = 0.000 and P = 0.001, respectively) (Fig. 5C,D).

## Discussion

Here, we investigated the analgesic effect of intrathecal injection of PDGF siRNA in a bone cancer pain animal model generated by injecting MRMT-1 tumor cells into the tibias of female rats. First, we developed a bone cancer pain animal model that mirrored features of clinical bone cancer pain^28^, including evoked and spontaneous pain. The number of tumor cells used for tibia injections (4 × 10^5^ cells), host immune suppression by daily cyclosporine administration, and the intrathecal delivery of the HSV virus carrying the PDGF siRNA were critical factors in establishing this bone cancer pain model^29^. Moreover, sterilized medical bone wax was used to close the injection site to prevent backrush of tumor cells after injection into the marrow cavity^30^. Female rats were used because they are more sensitive to pain stimuli and exhibit more pronounced behavioral effects^6^,^31^. Twenty-eight days after the injection of the tumor cells in the tibia, the animals developed thermal and mechanical hyperalgesia, as demonstrated by the reduction in TL and PWT. H&E staining revealed the presence of tumor cells and activated osteoclasts in the tibia, and radiography confirmed bone destruction. These characteristics of the bone cancer pain model are consistent with the findings of previous studies^32^,^33^.

Morphine, an opioid receptor agonist, is a commonly used clinical analgesic^34^, but its clinical use is limited by tolerance, addiction and adverse effects^35^. A single dose of morphine for analgesia does not affect GFAP expression or activate astrocytes in the spinal cord^36^,^37^, consistent with our recent data. Acute administration of morphine blocks spinal NK1r internalization (SP release) and abolishes the nerve-evoked release of SP^38^,^39^. Welch S P *et al* previously reported that acute administration of morphine suppresses the expression of CGRP in the corpus striatum^40^. Morphine treatment also markedly attenuated phosphorylation of ERK1/2, which could induce the hyperalgesia^41^. Therefore, the mechanisms by which morphine and PDGF siRNA induce analgesia may involve little kind of same identical molecules as the above described. In the present study, we compared the efficacy of PDGF siRNA and morphine in treating bone cancer pain. The mechanical and thermal hyperalgesia induced by the injections of tumor cells into the tibias of rats were reversed by RNAi-mediated inhibition of PDGF or by treatment with morphine. These results demonstrate that intrathecal administration of PDGF siRNA can alleviate pain associated with bone cancer.

Central sensitization is a type of activity-dependent plasticity in spinal cord neurons^42^ that results in hyperalgesia under pathological conditions^42^,^43^. In our model of bone cancer pain, we observed astrocyte hypertrophy and increases in the levels of SP, CGRP, pERK/ERK, and pAKT/AKT in the spinal cord segments L4–6, consistent with previous reports^44^,^45^. Astrocyte hypertrophy is observed in multiple inflammatory and neuropathic pain models^46^,^47^, suggesting that astrocytes partially mediate pain in these contexts^48^. Increased expression of SP is an indirect marker of sensitization of primary afferents^45^, whereas an increase in CGRP in laminae I-II of the spinal cord dorsal horn might be associated with the mechanisms underlying peripheral and spinal pain^49^. Our study provides novel evidence indicating that treatment with PDGF-siRNA attenuates astrocyte activation and reduces the expression of SP and CGRP in lamina I-II of the dorsal horn. These findings suggest that down-regulation of PDGF via treatment with PDGF siRNA is an effective strategy to attenuate the sensitization of the primary afferent centers in the spinal cord dorsal horn that are associated with the release of chemicals that mediate pain.

PDGF signaling contributes to the pathogenesis of neuropathic pain^26^. PDGF isoforms bind to PDGF receptors to induce receptor dimerization and autophosphorylation and, ultimately, the activation of downstream signaling pathways. The PDGF-B chain activates both PDGFRα and PDGFRβ^50^; therefore, we evaluated PDGFRα and PDGFRβ. The levels of phosphorylated PDGFRα and PDGFRβ were down-regulated in the PDGF siRNA-treated group. The ERK and AKT signaling pathways are frequently reported to be involved in pain and are verified therapeutic targets for treating diseases associated with pain^51^,^52^. The increased levels of pERK and pAKT in the bone cancer model evaluated in this study are in agreement with results from previous studies^53^,^54^ demonstrating that pERK and pAKT levels in the spinal cord increase under conditions of inflammation and neuropathic pain^55^,^56^. Phosphorylation of ERK has been reported as a marker of central sensitization^54^,^57^,^58^. Activation of the ERK and AKT signaling pathways is associated with the modulation of nociceptive information and peripheral and central sensitization in the spinal cord^53^,^59^. In the current study, intrathecal injection of PDGF siRNA inhibited ERK and AKT signaling, indicating that PDGF siRNA alleviated pain by decreasing the phosphorylation of PDGFRs and down-regulating the ERK-AKT signaling pathway, events that ultimately result in the down-regulation of GFAP, SP and CGRP expression.

In conclusion, RNAi-mediated down-regulation of PDGF inhibits bone cancer pain in the spinal cord. Therefore, reducing PDGF levels in the spinal cord using RNAi might be an effective therapeutic approach for relieving CIBP.

## Materials and Methods

### Induction of cancer and bone cancer pain

Animal care procedures and all experimental protocols were approved by the Sichuan Medical Experimental Animal Care Commission. The methods were conducted in accordance with the guidelines of the International Association for the Study of Pain^60^. A total of 70 female Sprague-Dawley rats (200–250 g) (provided by Department of Experimental Animal Center, Sichuan University) were used in this study. Throughout the experiments, 5–6 animals were housed in each cage with a 12-hour alternating light-dark cycle at 25 °C and free access to water and food. Ten animals were randomly assigned to the normal control group. Immunosuppression in the tumor-injected rats was initiated on the day of tumor cell injection via subcutaneous administration of cyclosporine (1 mg/100 g weight; 250 mg/5 mL, diluted in 50 mL of normal saline (NS); n = 60). Cyclosporine was administered daily for 28 days after the tumor cells were injected. The cell injection protocol used in this study has been previously described^61^. Briefly, 1 × 10^7^ mammary rat metastatic tumor (MRMT-1) cells (Haibo Whole Seoul Biotechnology Co., Lt) were harvested by centrifugation (10 min at 1,000 rpm) and diluted to a final concentration of 4 × 10^5^ cells/mL in NS solution. Anesthesia was administered via intraperitoneal injection of 3.6% chloral hydrate (1 mL/100 g), and 10 μL of a tumor cell solution was injected into the bilateral tibial bone cavity using a micro-syringe (n = 60). The pinhole was sealed with bone wax to prevent the carcinoma cells from leaking out along the injection track. Four weeks after the injection of tumor cells, PDGF expression in the spinal cord and bone destruction in the tibia were assessed by radiology and histological staining in 10 randomly selected rats. Subarachnoid catheterization was performed in the surviving rats (n = 32).

### Intrathecal insertion of the catheter at the lumbar 4–6 level of the spinal cord

We used a previously described procedure with some modifications^62^. Briefly, after anesthesia was administered, the fourth lumbar spinous process was removed, and the correct location was confirmed by puncturing the dura mater with a needle. After inserting a polyethylene catheter (PE-10, 15 cm) filled with NS 2cm into the subarachnoid space, the remaining portion of the catheter was sutured to the surrounding tissue to prevent slipping. More than 4 cm of the catheter was left outside the nape cervical skin, and the remainder was fixed and remained under the skin. The wound was closed in layers. Three days later, 10 μL of a 2% lidocaine solution was injected into each animal through the catheter. Animals that did not experience temporary paralysis of the hind limbs following lidocaine injection were excluded from the study. Ultimately, 22 rats were included for further analysis.

### HSV-1-based construct of the small interfering RNA targeting PDGF

The sequences of the PDGF siRNA expressed from the U6 promotor (21 bp) were as follows: rPDGFi-F1, agctGCAGGTGAGAAAGATCGAAATcgaaATTTCGATCTTTCTCACCTGCtttttAT, and rPDGFi-R1, CGATaaaaaGCAGGTGAGAAAGATCGAAATttcgATTTCGATCTTTCTCACCTGC. The sequences of the PDGF siRNA expression from the H1 promoter (19 bp) were as follows: rPDGFi-F2, GAGACTCCGTAGACGAAGAttcaagacaTCTTCGTCTACGGAGTCTCTTTTTAT and rPDGFi-R2, CGATAAAAAGAGACTCCGTAGACGAAGAtgtcttgaaTCTTCGTCTACGGAGTCTCgtac. These sequences were verified by chip-Seq technology. The 2 vectors used in the study were a non-replicating negative control HSV-1 (NC-siRNA) and pNX01-U6H1-rPDGFi (HSV-siPDGF). The vectors were co-transfected into the OG01 cell line, which expresses the ICP27 and ICP4 proteins required for the packaging of the virus harboring the PDGF siRNA. The titer of NC-siRNA and HSV-siPDGF injected was 5 × 10^9^ and 5.6 × 10^9^, respectively. The PDGF-B chain was specifically targeted by HSV-siPDGF in this study because expression of the PDGF-B chain is induced by peripheral nerve injury^63^.

### Drug Administration

Drugs were administered by connecting a 50-μL micro-syringe pre-filled with drug to the catheter. Morphine was dissolved in endotoxin-free physiological saline at a final concentration of 1 μg/mL for subarachnoid administration (20 μL; n = 5). Endotoxin-free physiological saline (n = 5), HSV-siPDGF (n = 6) and NC-siRNA (n = 6) were administered in equal volumes.

### Behavioral analysis

The latency of the response of the rat tail to a radiating thermal stimulus was measured using an automatic tail-flick analgesiometer (Ugo 7360, Italy) to indicate the degree of thermal hyperalgesia. Metal fixators were used to restrain the rat while their tails hung freely to receive the radiant heat source on the third caudal position. The intensity of the radiant heat source was 80 Hz and the cutoff time was 20 s. The test was performed after the animal became stable, i.e., the rat adapted to the restraining condition. Then, TL was automatically recorded when an abrupt flick of the tail occurred. To ensure consistency, each measurement was repeated 3 times in a blinded manner.

Mechanical hyperalgesia was measured by a paw withdrawal test using a Randall Selitto paw pressure device (Bioseb, France) that applied a linearly increasing mechanical force with an acrylic stylus. The stylus was placed on the middle of the dorsum of the rat’s paw. The PWT was recorded when the rat withdrew its paw. Data for both the left and right paws of each animal were collected and recorded in grams. Each measurement was repeated 3 times in a blinded manner. The data were averaged for statistical analysis.

The 2 behavioral tests were performed prior to surgery to determine the baseline values for each rat and 7, 14, 21 and 28 days after the tumor cell injections. Hyperalgesia was assessed on days 7 and 14 after intrathecal catheterization and gene administration for all groups, with the exception of the morphine-treated group, for which the tests were performed prior to the injection morphine and 10 min post-morphine injection at each indicated time point. The rats were acclimated for more than 30 min before the tests. To measure the threshold of mechanical and thermal hyperalgesia, the interval for each test was at least 10 min.

### Radiology

Destruction of the tibia bone was assessed 28 days after tumor cell injection. The hind limbs were isolated from the body, and the tibias were placed on Industrex X-ray film (Kodak, Italy) and exposed to an X-ray source (Faxitron) for 1 min at 30 kVp.

### Tissue preparation

Four weeks after tumor injection, the tibias of 10 randomly selected rats were isolated and immersed in 4% paraformaldehyde (PFA) for decalcification and histological staining. Two weeks later, the rats from each group were perfused and processed for immunofluorescence, western blot analysis and quantitative PCR (qPCR). After anesthetization, the animals were intracardially perfused with NS. Spinal cord segments L4-L6 were harvested. Half of the harvested spinal cords were fixed in 4% PFA, and the other half were frozen at −80 °C.

### Real-time qPCR

Quantitative PCR was performed as previously described^64^. PCR reactions were performed in a final volume of 20 μL containing 2x PCR master mix (Fermentas, K0171), sense and antisense primers, and diluted cDNA. The following primers were used to amplify PDGF (Gene ID: 14573): sense, AGGAAGATTCGTTCAGACCT; antisense, GTTCTCTTGGAGTCGCTCTG; probe sequence, CCAGTGGCAAGCACTGATC. The annealing temperature was 53 °C. qPCR amplification was performed using a Rotor-gene QTAMRA 1109 Sequence Detection System (QIAGEN Techservice) and the following reaction conditions: 2 min at 95 °C for polymerase activation and 95 °C for 15 s, 53 °C for 20 s, and 60 °C for 30 s for amplification and signal collection. *β-actin* was used to normalize the expression levels of target genes, and the −ΔΔCt method was used to evaluated differential expression.

### Immunofluorescence (IF)

PFA-fixed L4 spinal cord segments were embedded in O.C.T. compound (Tissue-Tek, Sakura, USA), and 10-μm serial transverse sections were sliced using in a cryostat. The sections were blocked with 5% normal goat serum for 2 h and incubated overnight at 4 °C in a humidified chamber with the following primary antibodies: rabbit polyclonal anti-PDGF BB (1:100) (ab16829, Abcam), mouse monoclonal anti-NeuN (1:400) (ab104224, Abcam), mouse monoclonal anti-GFAP (1:50) (MAB3402x, Millipore), mouse monoclonal anti-OX-42 (1:100) (MCA275G, AbDsreotec), rabbit polyclonal anti-RFP (1:400) (r10367, Abcam), rabbit polyclonal anti-SP (1:100) (AB1566, Chemicon) and rabbit polyclonal anti-CGRP (1:200) (ab22560, Abcam). The primary antibodies were dissolved in 2% normal goat serum. The sections were washed 3 times with PBS for 5 min each and incubated with the appropriate secondary antibodies: goat anti-rabbit (1:400) (A-24921, Invitrogen) and goat anti-mouse (1:400) (A-24920, Invitrogen) for 2 h at 37 °C. The sections were washed with PBS 3 times for 5 min each, stained with DAPI and mounted. To confirm the specificity of the PDGF labeling, the synthetic immunogen (HangZhou HuaAn Biotechnology Co. Ltd) was pre-incubated with the PDGF-BB antibody. Then, sections in this experimental control were performed by the same process as described above (see Supplementary Fig. S1).

### Quantification of immunofluorescence

Immunofluorescence was evaluated in the spinal cord at the lumbar 4 (L4) level because L4 is one of the main projection sites of primary afferent fibers innervating the hind limbs^45^,^65^. The saline- and morphine-treated groups included 5 animals each, and the NC- and HSV-siPDGF-treated groups included 6 animals each. Immunofluorescence intensity was measured from images captured by a Leica DMI 6000B fluorescence microscope. SP and CGRP immunoreactivity was quantified in laminae I–II, and GFAP immunofluorescence intensity was analyzed in laminae I-V of the dorsal horn in 5 randomly selected L4 sections per mouse. Briefly, 400× images of the dorsal horn of the spinal cord were acquired using an exposure time of 150 ms. Three independent experiments using the same method and software (Image J) were performed, after background subtraction, the mean fluorescence gray intensity of each image was determined. The results are presented as the mean gray intensity values of GFAP, CGRP and SP immunofluorescence normalized to normal.

### Histological staining

Tibia bones were fixed in 4% PFA for 1 week, and decalcified in PFA containing 5% formic acid for an additional 4 weeks. Prior to wax embedding, tibia bone tissue was rinsed and dehydrated. 5-μm longitudinal sections were cut from the paraffin-embedded blocks using a rotary microtome, and the sections were stained with hematoxylin and eosin to visualize the extent of tumor infiltration and bone loss.

### Western blot (WB)

Total protein was extracted from the L4-L6 spinal cord segments as previously described^66^. Proteins were separated by SDS-PAGE and transferred to PVDF membranes. The membranes were incubated overnight at 4 °C with the following primary antibodies: rabbit anti-GFAP (1:500) (ab53554, Abcam), rabbit anti-Substance P (1:500) (orb215527, Biorbyt), rabbit anti-CGRP (1:500) (ab47027, Abcam), rabbit anti-PDGF BB (1:300) (ab16829, Abcam), rabbit anti-pPDGFRα (1:1000) (#2992, Cell Signal), rabbit anti-pPDGFRβ (1:1000) (#2227,Cell signal), rabbit anti-ERK(1:500) (ab184699, Abcam), rabbit anti-pERK (1:1000) (#4370, Cell Signal), rabbit anti-AKT (1:500) (9272s, Cell Signal) and rabbit anti-pAKT (1:1000) (4056s, Cell Signal). The membranes were subsequently incubated with the appropriate secondary antibodies (1:5000; Abcam) for 1 h at room temperature. The protein bands were detected using an enhanced chemiluminescence detection system. Then, the membranes were stripped with stripping buffer (P0025, beyotime) and reblotted with antibodies against the loading controls [mouse anti-β-actin (1:5000) (ab119716, Abcam) and mouse anti-GAPDH (1:5000) (ab9484)]. The intensity of the immunoreactive bands was quantified using Image J software. The ratio of target protein to *β-actin* (loading control) levels was statistically analyzed. Western blot experiments were repeated 3 times.

### Statistical analyses

All data were recorded as the mean ± standard error of the mean (SEM), and statistical analysis was performed using SPSS 16.0 software. The statistical significance of differences between groups was analyzed with a one-way analysis of variance (ANOVA) and the Bonferroni/Dunn multiple-comparison test. P < 0.05 was considered statistically significant.

## Additional Information

**How to cite this article**: Xu, Y. *et al* Mechanisms of PDGF siRNA-mediated inhibition of bone cancer pain in the spinal cord. *Sci. Rep.* **6**, 27512; doi: 10.1038/srep27512 (2016).

## Supplementary Material

Supplementary Information
