# Supplementary material for: Mechanisms of PDGF siRNA-mediated inhibition of bone cancer pain in the spinal cord
Source: Sci Rep. 2016 Jun 10;6:27512. doi: 10.1038/srep27512 (PMC4901320; doi:10.1038/srep27512)
Supplement: Supplementary Information [file srep27512-s1.pdf]

Mechanisms of PDGF siRNA-mediated inhibition of bone cancer pain in the spinal cord

Yang Xu[1*], Jia Liu [2*], Mu He [3], Ran Liu[1], Visar Belegu[4], Ping Dai[3], Wei Liu[1], Wei Wang[1], Qing-Jie Xia[1], Fei-Fei Shang[1], Chao-Zhi Luo[1], Xue Zhou[5], Su Liu[4], JohnW.McDonald[4], Jin Liu[1], Yun-Xia Zuo[1], Fei Liu[1#]& Ting-Hua Wang[1,2#]

[1] Institute of Neurological Disease, Department of Anesthesiology and Translation Neuroscience Center, West China Hospital, Sichuan University, Chengdu 610041, PR China, [2] Institute of Neuroscience, Kunming Medical University, Kunming 650031, PR China, [3] Department of Neurosurgery, West China Hospital, Sichuan University, Chengdu 610041, PR China, [4]Department of Neurology, Johns Hopkins School of Medicine, Baltimore, MD, USA, International Center for Spinal Cord Injury, Hugo W. Moser Research Institute at Kennedy Krieger Inc., Baltimore, MD, USA, [5]Department of Histology, Embryology and Neurobiology, West China School of Preclinical and Forensic Medicine, Sichuan University, Chengdu, Sichuan 610041, China.

*, # These authors contributed equally to this work.

Correspondence to Ting-Hua Wang [tinghua_neuron@263.net]

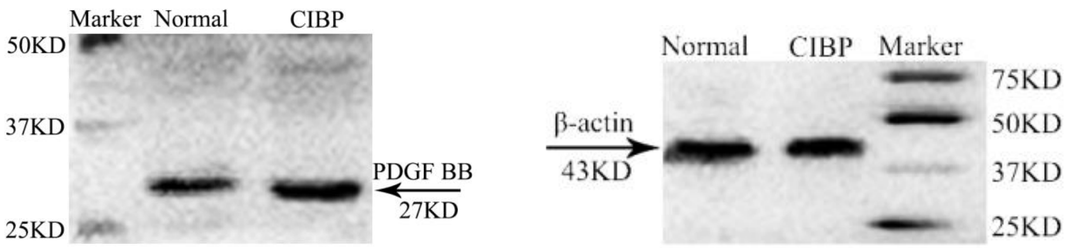

Full-length blots in Figure 1D

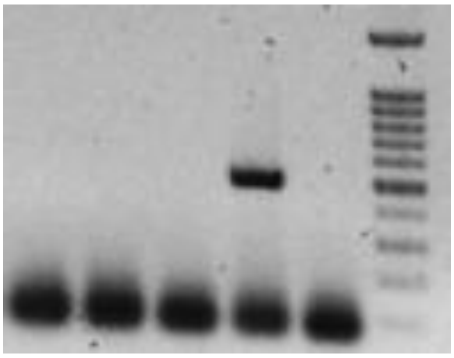

Full-length gels of detected the HSV-1-based virus using RT-PCR analysis in Figure 3C.

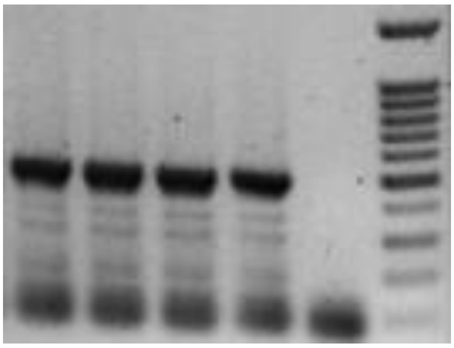

Full-length gels of detected of the HSV-1-based recombination using RT-PCR analysis in Figure 3B.

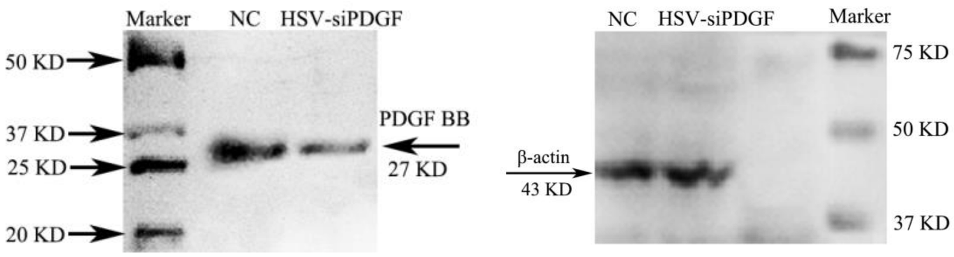

Full-length blots in Figure 4A

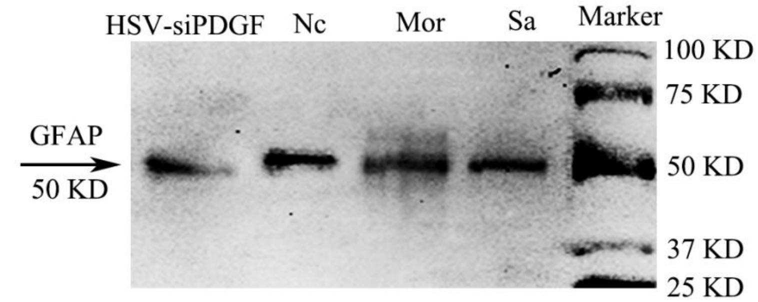

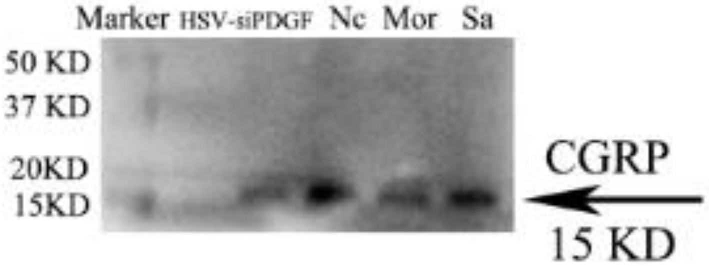

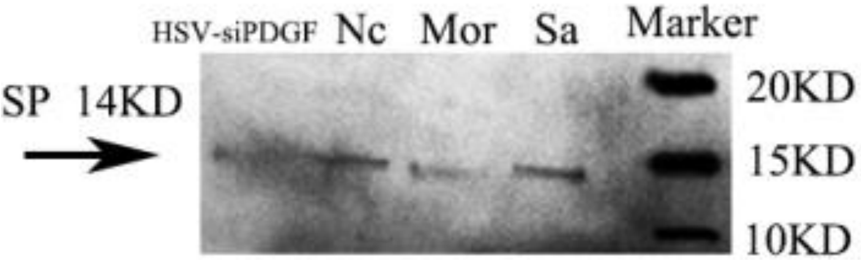

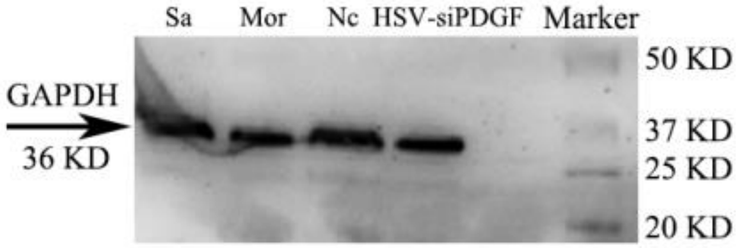

Full-length blots in Figure 4J

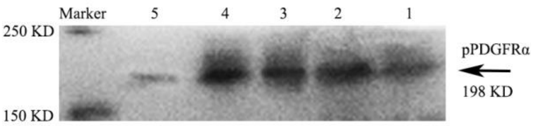

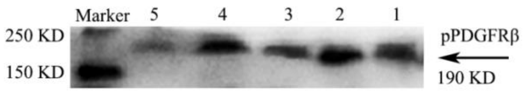

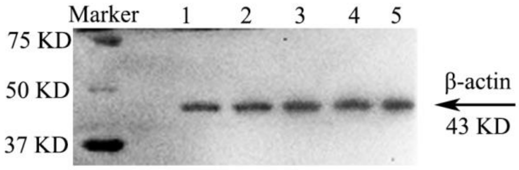

Full-length blots in Figure 5A. Lane 1: Normal; Lane 2：Treatment with saline; Lane 3: Treatment with morphine; Lane 4: Treatment with negative control siRNA; Lane 5: Treatment with PDGF siRNA.

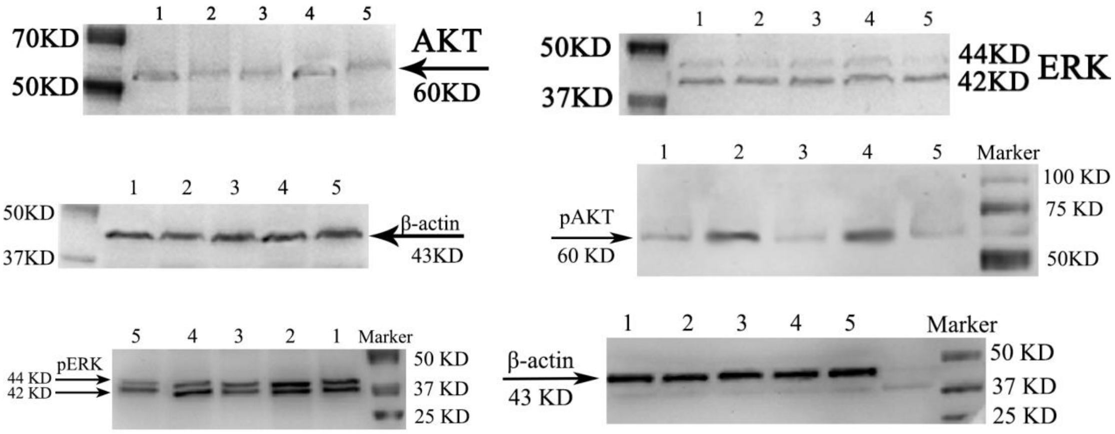

Full-length blots in Figure 5C. Lane 1: Normal; Lane 2：Treatment with saline; Lane 3: Treatment with morphine; Lane 4: Treatment with negative control siRNA; Lane 5: Treatment with PDGF siRNA.

Data information for paw withdrawal thresholds in Figure 4B

Data are presented as the mean±SEM (0 h after surgery: 9.4±0.57 in the saline group, 8.1±0.99 in the morphine group, 9.0±1.53 in the negative control group, 9.0±1.33 in the HSV-siPDGF group;1 week after surgery: 11.2±1.49 in the saline group, 8.7±1.45 in the morphine group, 9.3±1.72 in the negative control group, 8.3±1.22 in the HSV-siPDGF group; 2 week after surgery: 9.5±3.48 in the saline group, 7.2±2.01 in the morphine group, 8.0±1.55 in the negative control group, 8.5±1.97 in the HSV-siPDGF group; 3 week after surgery: 11.0±2.13 in the saline group,

6.8±0.86 in the morphine group, 6.2±1.13 in the negative control group, 8.3±1.55 in the HSV-siPDGF group; 4 week after surgery: 6.3±1.53 in the saline group, 6.7±1.81 in the morphine group, 6.4±1.97 in the negative control group, 5.6±1.22 in the HSV-siPDGF group; 1 week after injection: 6.5±0.50 in the saline group, 10.2±0.94 in the morphine group, 8.6±3.63 in the negative control group, 8.3±1.27 in the HSV-siPDGF group; 2 week after injection: 6.6±0.48 in the saline group, 9.5±0.65 in the morphine group, 7.0±0.89 in the negative control group, 9.0±0.78 in the HSV-siPDGF group).

Data information for tail-flick latencies in Figure 4C

Data are presented as the mean ± SEM (0 h after surgery: 9.0±1.13 in the saline group, 8.0±0.48 in the morphine group, 9.0±1.53 in the negative control group, 9.0±1.11 in the HSV-siPDGF group; 1 week after surgery: 7.2±0.85 in the saline group, 7.3±0.78 in the morphine group, 8.2±1.55 in the negative control group, 7.9±1.21 in the HSV-siPDGF group; 2 week after surgery: 6.7±1.56 in the saline group, 6.3±1.20 in the morphine group, 7.1±0.67 in the negative control group, 7.9±0.62 in the HSV-siPDGF group; 3 week after surgery: 4.7±0.80 in the saline group, 5.9±0.63 in the morphine group, 5.9±0.88 in the negative control group, 5.9±0.94 in the HSV-siPDGF group; 4 week after surgery: 3.3±0.21 in the saline group, 4.0±0.46 in the morphine group, 3.8±0.34 in the negative

control group, 4.7±3.08 in the HSV-siPDGF group; 1 week after injection: 5.2±1.85 in the saline group, 9.18±0.94 in the morphine group, 4.1±1.02 in the negative control group, 10.0±0.53 in the HSV-siPDGF group; 2 week after injection: 5.5±1.71 in the saline group, 9.6±0.62 in the morphine group, 4.3±0.61 in the negative control group, 8.5±1.65 in the HSV-siPDGF group).

Supplemental Figure Legends

Supplementary Figure 1. The specificity of the PDGF labeling is confirmed by the results of experimental controls. The immunogen of PAGF-BB antibody (Abcam, ab16829) were synthesized by HangZhou HuaAn Biotechnology Co. Ltd (Wensan road 199, Hangzhou, China. http://old.huabio.com/en/index.asp). The amino acid sequence of this peptide is TEVFEISRRLIDRTNA, which are corresponding to amino acids 101-116 of PDGF BB. This peptide had a purity of >95%. The characterization and purity estimation of the peptides were performed by using electrospray mass spectrometry (ESMS) and reverse phase high performance liquid chromatography (RP-HPLC). All peptides were dissolved in sterilized water to 5.6 mg/ml and stored in aliquots at 4 ℃. Briefly, the PDGF-BB antibody was pre-incubated with the synthetic peptide (1:5) overnight at 4°C. The ratio was supported by the customer technical support engineer of Abcam in China. Then，the IF experiment

was done as described. The upper panel was stained with rabbit polyclonal anti-PDGF BB showing obvious PDGF immunofluorescence, whereas the images in the lower panel representing the results of the experimental control did not show any immunoreactivity.

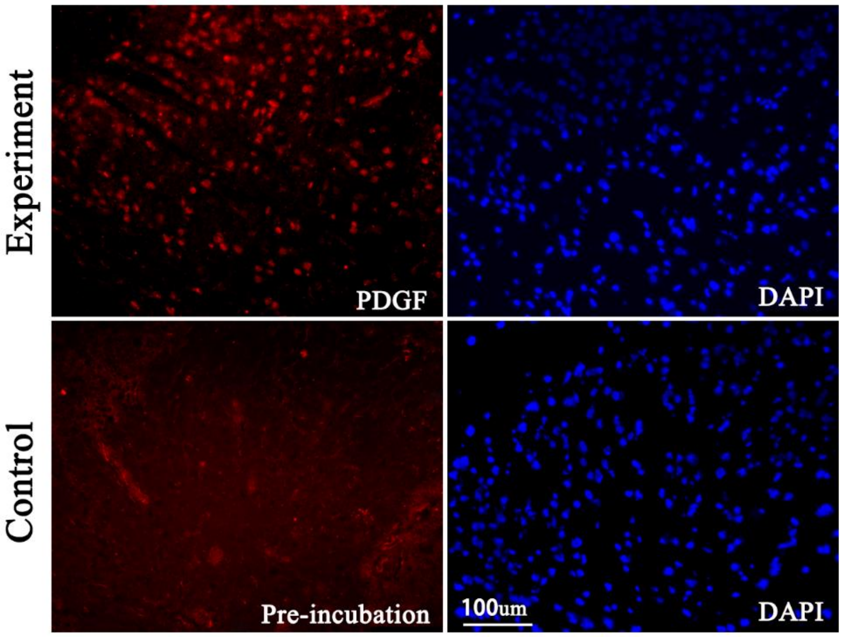

Supplement Fig. S1
